# Unusual angiographic images of unruptured, large, kissing middle cerebral artery aneurysms

**DOI:** 10.1007/s00701-012-1555-2

**Published:** 2012-11-23

**Authors:** Bogdan Czapiga, Marta Kozba-Gosztyla, Slawomir Bereza, Wlodzimierz Jarmundowicz

**Affiliations:** 1 Department of Neurosurgery, Wroclaw Medical University, Borowska Street 213, 50-556 Wroclaw, Poland; 2 Department of Radiology, Wroclaw Medical University, Wroclaw, Poland

Dear Editor,

Kissing aneurysms, defined as two adjacent aneurysms of the same or different arteries with separate origins and partially adherent walls, are rare findings with an incidence of less than 1 % [3, 8]. The most uncommon site of their occurrence is a middle cerebral artery (MCA). An extensive literature search revealed only two cases reported in this location [1, 7]. We present a case of kissing, unruptured, large MCA aneurysms with unusual angiographic images that resulted in substantial diagnostic difficulties.

**Fig. 1:**
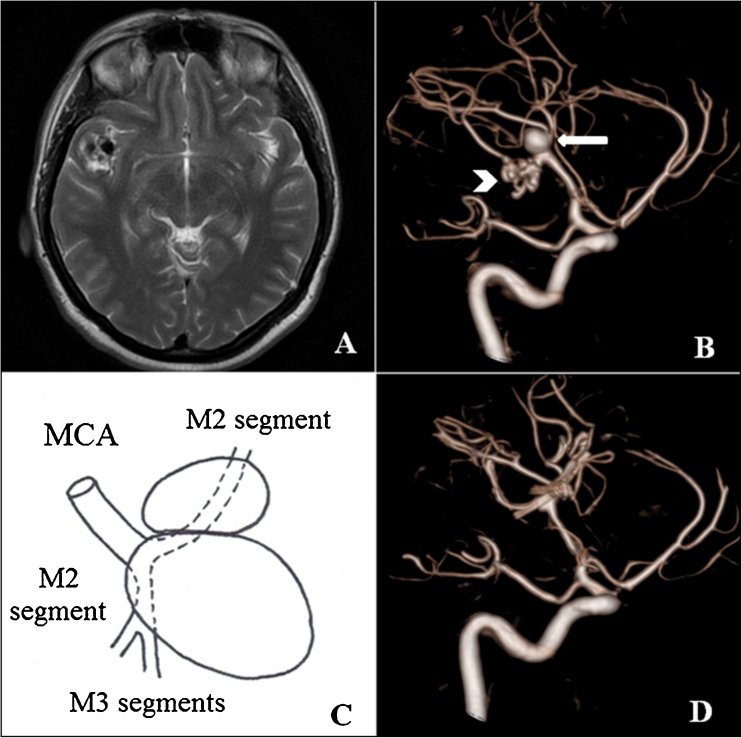
**a** Preoperative T2-weighted MR image showing a pathological mass in the location of the first right MCA bifurcation. **b** Right internal carotid artery rotational 3D DSA showing saccular aneurysm (*arrow*) with network of tortuous, segmentally ectatic vessels (*arrowhead*). **c** Schematic drawing showing kissing aneurysms and the arrangement of feeding vessels. **d** Postoperative angiogram revealing successful obliteration of the two aneurysms with preservation of normal blood flow through the right middle cerebral artery and all of its branches

A 45-year-old female was referred to magnetic resonance imaging (MRI) because of hemifacial pain. MRI revealed a well-defined, regular mass in the location of the first right MCA (rMCA) bifurcation with a diameter of 33 mm. The lesion had a mixed signal in both T1- and T2-weighted images with a presence of methemoglobin and irregular, flow-void regions inside with vascular type of contrast enhancement (Fig. 1a), which suggested a giant, multilobed MCA aneurysm. On admission to our department, the patient was asymptomatic, with the hemifacial pain resolving spontaneously. The cerebral digital subtraction angiogram (DSA) with three-dimension (3D) rotational acquisition showed a saccular aneurysm (0.6 × 0.7 cm) of rMCA bifurcation. Additionally, a network of tortuous, segmentally ectatic vessels, replacing proximal 2 cm of the angular artery was seen. The angiographic findings were interpreted as segmental agenesis of right angular artery and a *rete mirabile* type of network, with ectatic and aneurysmal changes (Fig. 1b). Taking into account the patient’s relatively young age, the size and location of aneurysm and the presence of a *rete mirabile* type of network, the multidisciplinary team’s consensus was to proceed with a surgical treatment. We decided to use intraoperative monitoring of somatosensory and motor evoked potentials. Our plan for surgery was to clip the aneurysm that was more superficial and then after placing a temporal clip on a feeding vessel of the pathological network, without detectable changes in evoked potentials, to close it. We proceeded with the right pterional craniotomy. After exposing the Sylvian fissure, the large partially thrombosed aneurysm was demonstrated. It was arising from the M2 segment, right behind the first MCA bifurcation with a diameter significantly greater than that estimated by DSA (more than 20 mm). More profoundly, there was another aneurysm, similarly partially thrombosed, and smaller than the first one (more than 10 mm). It originated from the second M2 segment and was misinterpreted preoperatively as a *rete mirabile* type of network (Fig. 1c). The aneurysms had partially adherent walls that were separated in the first stage. Next, under the temporary occlusion of the main MCA branch (7 min, 35 s), the sacks of both aneurysms were opened and cleaned from thrombi to enable appropriate clip placement (two clips for each one). During the procedure, no changes were observed in the evoked potentials. The patient had an uneventful recovery. Control DSA performed 1 month after surgery revealed a small (3 mm) residual aneurysm with restoration of anatomic lumen of the right angular artery as well as complete occlusion of proximal aneurysm located in the first rMCA bifurcation with preservation of normal blood flow through rMCA and all of its branches (Fig. 1d).

The preoperative diagnosis of multiple or kissing aneurysms is often difficult. They can easily be misinterpreted as a single multilobed aneurysm or a vessel’s wall irregularity due to atherosclerotic arterial changes and result in an unpredictable finding during surgery [5]. According to Harada et al., 57 % of kissing aneurysms were not recognized preoperatively [1]. Special angiographic projections and 3D DSA, which are nowadays widely used, are thought to reduce these problems [2]. However, in our case, DSA results were misleading and an intraoperative finding of kissing aneurysms, instead of one aneurysm with coexisting pathological network, was unexpected. Our treatment was reviewed by the multidisciplinary team which concluded that if the preoperative correct diagnosis would have been established, the treatment method would have been the same, although there are some reports about successfully endovascular treatment of kissing aneurysms [4, 6].

The authors thank Anna Usowicz MD from the Children’s Hospital Oakland and Research Institute for her cooperation and assistance in editing the article.
